# *cmpX* overexpression in *Pseudomonas aeruginosa* affects biofilm formation and cell morphology in response to shear stress

**DOI:** 10.1016/j.bioflm.2024.100191

**Published:** 2024-03-15

**Authors:** Audrey David, Mélissande Louis, Ali Tahrioui, Sophie Rodrigues, Clarisse Labbé, Olivier Maillot, Magalie Barreau, Olivier Lesouhaitier, Pierre Cornelis, Sylvie Chevalier, Emeline Bouffartigues

**Affiliations:** a Univ Rouen Normandie, Université Caen Normandie, Normandie Univ, CBSA UR 4312, F-76000, Rouen, France; b Laboratoire de Biotechnologie et Chimie Marines, Université Bretagne Sud, EMR CNRS 6076, IUEM, 56100, Lorient, France

**Keywords:** CmpX, Biofilm, Dynamic flow

## Abstract

*Pseudomonas aeruginosa* is an opportunistic pathogen causing chronic infections that are related to its ability to form biofilms. Mechanosensitive ion channels (Mcs) are cytoplasmic membrane proteins whose opening depends on a mechanical stress impacting the lipid bilayer. CmpX is a homologue of the small conductance MscS of *Escherichia coli*. The *cmpX* gene is part of a transcriptional *cfrX-cmpX* unit that is under the control of the cell envelope stress response ECF sigma factor SigX. CmpX was shown to regulate the activity of the hybrid sensor kinase PA1611 involved in the regulation of transition from a planktonic to a biofilm lifestyle. The deletion of *cmpX* leads to increased biofilm formation under static conditions. Herein, the effect of *cmpX* overexpression was investigated by confocal laser scanning microscopy in terms of biofilm formation and architecture, and matrix components production, in dynamic conditions. We show that overexpression of *cmpX* in *P. aeruginosa* leads to enhanced and altered biofilm architecture that seems to be associated to increased matrix components and the emergence of filamentous cells. These phenotypic alterations might occur potentially through a shear stress induced by the medium flow rate.

**Importance:**

CmpX is involved in biofilm formation and cell filamentation with regards to the medium flow.

## 1 Introduction

*Pseudomonas aeruginosa* is a highly widespread adaptable Gram-negative bacterium that can colonize a broad of hosts, including insects, plants, and mammals. In humans, *P. aeruginosa* is an opportunistic pathogen causing severe acute and chronic infections notably in cystic fibrosis-suffering patients and immunocompromised patients. Colonization of *P. aeruginosa* in chronic infections is related to the formation of microbial communities, the so-called biofilms, which are difficult to eradicate, and are a major cause of antibiotic tolerance [1].

Bacteria are responsive to environmental changes, during which they have to deal with fluid flow, osmotic pressure and contact with surfaces or other cells [2], generating significant mechanical stresses on the envelope. Such stresses then lead to an appropriate stress response allowing survival [3,4]. In particular, bacteria have developed mechanosensitive ion channels to reduce the cell turgor applied on cell membranes during the switch from high to low osmolarity, serving as “emergency valves” to avoid cell disruption and death [5]. Indeed, these cytoplasmic membrane gated proteins allow release of cytoplasmic solutes in response to membrane tensions generated by rapid water flow into the cell during an hypoosmotic shock [[5], [6], [7]]. The mechanosensitive channels of small conductance MscS, and of large conductance MscL, are the two major family players in the adaptation to environmental osmolarity. MscS opens at low membrane tensions, and MscL, at higher membrane perturbations [5,8,9]. While MscS has been widely studied in *Escherichia coli*, few data were reported in case of *P. aeruginosa*. However, nine proteins were predicted to belong to the Msc family in *P. aeruginosa,* among which one was proposed to belong to the large conductance MscL family, and eight to the MscS family [10,11]. In line with their involvement as sensors of membrane tensions, these proteins were suggested to be part of the cell envelope stress response (CESR) network, since some of them were regulated by the extracytoplasmic function sigma factors (ECFσ) AlgU or SigX [11,12]. For example, expression of the genes encoding the MscL homologue PA4614 and the MscS homologues PA1408, PA5125 and PA4394, was modulated in *algU* mutant- and -overexpressing strains [13,14].

CmpX (PA1775) is homologous to the *E. coli* MscS, and its encoding gene *cmpX* is part of the operonic structure *cfrX-cmpX*, located directly upstream of *sigX* [12]. SigX is a CESR ECFσ, which is involved in biofilm formation, cell envelope homeostasis, and virulence in *P. aeruginosa* [11,[15], [16], [17], [18], [19], [20]]. SigX was shown to regulate *cmpX* expression, notably in stress conditions leading to membrane fluidity alterations [12,[20], [21], [22], [23], [24]]. Transcription of *sigX* itself was reduced in a Δ*cmpX* mutant [25]. A transposon inserted in *cmpX* led to activate the expression of the hybrid histidine kinase PA1611 [26], a protein of the Gac-Rsm regulatory network, which is involved in the switch from a planktonic to a sessile lifestyle. In addition, CmpX was suggested to be involved in motility and biofilm formation, since a Δ*cmpX* mutant led to reduced swarming and twitching motilities, and increased biofilm formation by 1.5-fold in static conditions [25]. Altogether, these data suggest important functions of CmpX in the physiology and pathogenicity of *P. aeruginosa*. Herein, the effect of *cmpX* overexpression was investigated in dynamic conditions on biofilm formation and architecture, and matrix components production, by confocal laser scanning microscope (CLSM). Since cell morphology was affected by *cmpX* overexpression, the effect of the medium flow rate was also investigated on the biofilm architecture and cell morphology.

## 2 Results

***cmpX* overexpression leads to increased biofilm formation**. In *P. aeruginosa*, CmpX was previously shown to be involved in biofilm formation based on the results obtained from a *cmpX* mutant, using the crystal violet staining assay [25]. To get further insights, we constructed a *cmpX* overexpressing strain (H103*-*cmpX) and its isogenic control strain containing the empty vector (H103-EV). Both strains (H103*-*cmpX and H103-EV) were allowed to develop their biofilms for 24 h in dynamic conditions (3 mL h^−1^), in LB supplemented with 0.2% arabinose to induce expression of *cmpX* in H103*-*cmpX strain. Total RNAs were extracted from biofilms in these conditions and the RT-qPCR assays showed an increase in *cmpX* transcription by 7.4-fold in the *cmpX* overexpressing strain compared to the isogenic wildtype strain (Table 1). The biofilms formed were observed by CLSM. The images were analyzed using COMSTAT2 software. *P. aeruginosa* H103-EV displayed a flat and homogeneous biofilm architecture, while H103*-*cmpX formed a heterogenous biofilm structure (Fig. 1A). As shown by COMSTAT2 analysis (Fig. 1B), overexpression of *cmpX* significantly increased biofilm formation in terms of biovolume (by 2.5-fold), average (by 2.1-fold) and maximal thicknesses (by 2-fold). These data show that overexpression of *cmpX* affect biofilm formation and its architecture in dynamic flow cell conditions.

**Table 1:** Genes up- and down-regulated in H103*-*cmpX versus H103-EV. ***, P = 0.0001 à 0.001; **, P = 0.001 à 0.01.

Gene number	Gene name	Product name and/or function	Fold change
**PA1775**	*cmpX*	MscS homologue	7.4***
**PA1776**	*sigX*	ECF sigma factor	1.1
**PA1774**	*cfrX*	CfrX	1.3
**PA0762**	*algU*	ECF sigma factor	1.7
**PA3540**	*algD*	GDP-mannose 6-dehydrogenase	0.7
**PA2232**	*pslB*	PslB	1.2
**PA3063**	*pelB*	PelB	2.5**
**PA4625**	*cdrA*	Cyclic diguanylate-regulated TPS partner A	2.8**
**PA1181**	*PA1181*	Diguanylate cyclase	2.3**
**PA4407**	*ftsZ*	Cell division protein	2.7**

**Fig. 1:**
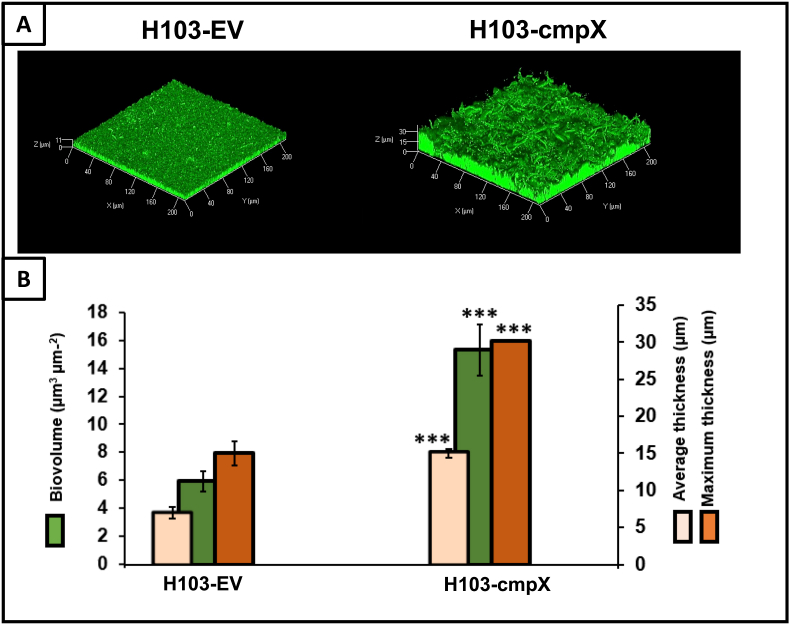
**Overexpression of *cmpX* leads to increased biofilm formation. (A)** CLSM images 3D of 24 h biofilms after biomass labelling by SYTO 9 green in H103-EV and H103*-*cmpX. The two strains were grown with 0.2% arabinose. Images show representative data from five independent biofilm assays. **(B)** COMSTAT2 analyses were performed to determine maximum thicknesses (μm), average thicknesses (μm), and biovolumes (μm^3^ μm^−2^). The error bars represent the standard error of the means (SEMs) and are the result of the analysis of three views of each of the five independent biological assays. Statistics were achieved by student's *t*-test between H103-EV vs H103*-*cmpX: ***, P = 0.0001 to 0.001; without stars: not significant, P ≥ 0.05. (For interpretation of the references to colour in this figure legend, the reader is referred to the Web version of this article.)

***cmpX* overexpression leads to cell filamentation within the biofilm.** In addition to increased biofilm formation, we noticed that cell morphology was strongly altered when *cmpX* was overexpressed, since numerous single and elongated thin cells were observed in biofilm cultures of the supplemented strain (H103*-*cmpX) versus the control one (H103-EV) (Fig. 2A). Thus, it seems that *cmpX* overexpression triggers a morphological change of certain biofilm cells leading to filamentation. We then questioned if cell filamentation was associated to biofilm lifestyle or if it occurs also in the planktonic state. H103-EV and H103-cmpX were grown in liquid LB medium to exponential and stationary phases. Supplementary Fig. S1 shows that no elongated cells were observed for both strains in each of these conditions. In addition, no filamentation was observed when the cells were allowed to adhere to a glass slide for 2 h (Supplementary Fig. S2). Altogether, these data suggest that filamentation was related to the biofilm lifestyle. Since filamentation is generally described as a stress response [27], we suspected that *cmpX* overexpression could be toxic in our condition. In planktonic condition, the growth curves of H103-EV and H103*-*cmpX were similar suggesting that the overexpression of *cmpX* did not affect the growth kinetic of *P. aeruginosa* (Supplementary Fig. S3). Within the biofilm, overexpression of *cmpX* led to a slight, but significant, increase of the proportion of injured cell (Fig. 2A). COMSTAT2 analysis (Fig. 2B) indicated the presence of about 5.6% of permeabilized cells by the red propidium iodide fluorescent dye and 94.4% of alive cells (green labeling). Noticeably, a sub-population of the elongated cells was labeled with propidium iodide, suggesting that they were permeabilized (Fig. 2A). Noticeably, most of the elongated cells that were observed in the lower part of the biofilm (Fig. 2C, S = 1), or in its middle part (Fig. 2C, S = 14), were labeled with propidium iodide, suggesting that these cells were damaged. The SYTO9-labeled filamentous cells were for most of them located into the middle (Fig. 2C, S = 14) and the upper parts of the biofilm (Fig. 2C, S = 25), suggesting that these cells were alive. As previously indicated, no such elongated cells were observed within the biofilm of H103-EV, neither at the lower, middle or upper parts (Fig. 2C, S = 1, S = 6 or S = 12). The expression of *sigX* or a*lgU*, encoding the CESR ECFσ SigX and AlgU respectively, and of their target genes *cfrX* and *algD*, was not affected in the H103*-*cmpX strain as compared to H103-EV (Table 1), indicating that the CESR was not affected by *cmpX* overexpression in our conditions. Altogether, these data suggest that overexpression of *cmpX* leads to increased biofilm formation with an altered architecture. Moreover, *cmpX* overexpression triggers to cell filamentation, and to a lesser extent, to cell damage within the biofilm.

**Fig. 2:**
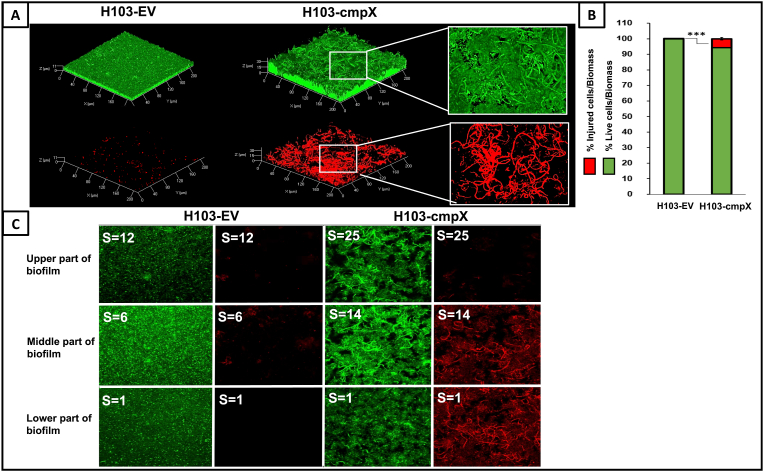
**Overexpression of *cmpX* leads to cell filamentation. (A)** CLSM images 3D of 24 h biofilms after biomass labelling by SYTO 9 green and propidium iodide (red) in H103-EV and H103*-*cmpX. The two strains were grown with 0.2% arabinose. Images show representative data from five independent biofilm assays. **(B)** COMSTAT2 analyses were performed to determine biovolume of live cells (green) or injured cells (red). The error bars represent the standard error of the means (SEMs) and are the result of the analysis of three views of each of the five independent biological assays. Statistics were achieved by student's *t*-test between H103-EV vs H103*-*cmpX: ***, P = 0.0001 to 0.001; without stars: not significant, P ≥ 0.05. **(C)** CLSM stack images of 24 h biofilms after biomass labelling by SYTO 9 (green) and propidium iodide (red) in H103-EV and H103*-*cmpX. S = Stack number; S1: lower part of the biofilm of H103-EV and H103-cmpX; S6 and 14: middle part of the biofilm of H103-EV and H103-cmpX, respectively; S12 and S25: upper part of the biofilm of H103-EV and H103-cmpX, respectively. (For interpretation of the references to colour in this figure legend, the reader is referred to the Web version of this article.)

***cmpX* overexpression leads to increased production of matrix components.** In *P. aeruginosa*, extracellular DNA (eDNA), alginate, Pel and Psl exopolysaccharides (EPS), and exoproteins are important matrix-components that maintain the biofilm architecture [[28], [29], [30]]. Prior to image acquisitions, eDNA, EPS, and exocellular proteins were labeled using the 7‐hydroxy‐9H‐(1,3‐dichloro‐9,9‐dimethylacridin‐2‐one) (DDAO), CalcoFluor White (CFW), and SYPRO Ruby fluorescent dyes, respectively. Cells within the biofilm were labeled using the green fluorescent SYTO9 dye. As shown on Fig. 3A, *cmpX* overexpression increases eDNA, EPS and exocellular proteins content. COMSTAT2 analyses of CLSM images, in which matrix components were normalized to biofilm biomass, confirmed the enhancement of the abundance of eDNA, EPS and exoproteins in H103-cmpX by about 8.7-fold, 10.3-fold and 7.6-fold, respectively, as compared to the control H103-EV strain (Fig. 3B). These data suggest that biofilm stimulation in the *cmpX* overexpressing strain could be associated to an increase of the matrix components production. The expression of several genes encoding enzymes of the alginate (*algD*), Pel (*pelB*) and Psl (*pslB*) EPS biosynthetic pathways were assayed using RT-qPCR assays (Table 1). Only *pelB* shows a significant increased transcription by about 2.5-fold in the *cmpX* overexpressing strain. CdrA is a major matrix protein that crosslinks Pel EPS to ensure biofilm architecture. CdrA expression is regulated by the second messenger bis-(3′–5′)-cyclic dimeric guanosine monophosphate (c-di-GMP), the concentration of which is tightly correlated to increased biofilm formation [31,32]. In our conditions, *cdrA* expression was increased by 2.8-fold in the H103*-*cmpX as compared to H103-EV, as well as the diguanylate cyclase encoding gene PA1181, which is involved in c-di-GMP production [20].

**Fig. 3:**
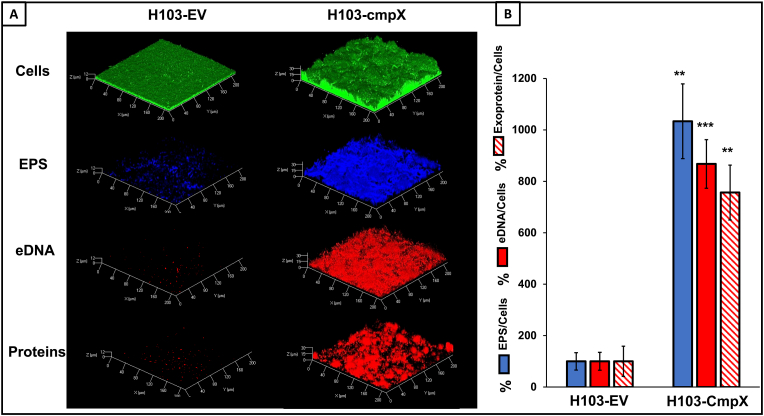
**Overexpression of *cmpX* leads to increased matrix components. (A)** CLSM images 3D of 24 h biofilms after biomass labelling by SYTO 9 (green), EPS by calcofluor white (blue), eDNA by DDAO and matrix proteins by SYPRO Ruby (red) in H103-EV and H103*-*cmpX grown with 0.2% arabinose. Images show representative data from three independent biofilm assays. **(B)** COMSTAT2 analyses were performed to determine biovolume of EPS (blue), eDNA (red) and matrix proteins (red hatches). The error bars represent the standard error of the means (SEMs) and are the result of the analysis of three views of each of the three independent biological assays. Statistics were achieved by student's *t*-test between H103-EV and H103*-*cmpX: ***, P = 0.0001 to 0.001; **, P = 0.001 to 0.01; without stars: not significant, P ≥ 0.05. (For interpretation of the references to colour in this figure legend, the reader is referred to the Web version of this article.)

**Fig. 4:**
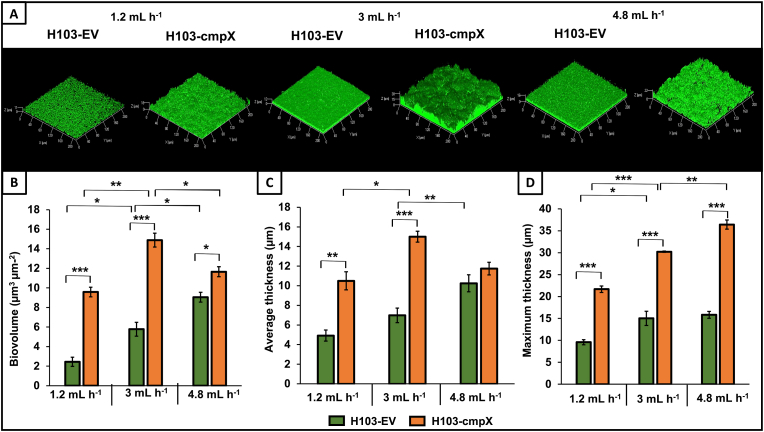
**Effect of flow rates on biofilm formation and architecture.** CLSM images 3D of 24 h biofilms after biomass labelling by SYTO 9 (green) at 1.2 mL h^−1^, 3 mL h^−1^ and 4.8 mL h^−1^ in H103-EV and H103*-*cmpX. Images show representative data from three independent biofilm assays. **(A)** COMSTAT 2 analyses were performed from CLSM images to determine biovolume **(B)**, average thickness **(C)**, and maximum thickness **(D)**. The error bars represent the standard error of the means (SEMs) and are the result of the analysis of three views of each of the three independent biological assays obtained by CLSM assays. Statistics were achieved by student's *t*-test between H103-EV and H103*-*cmpX: ***, P = 0.0001 to 0.001; **, P = 0.001 to 0.01; *, P = 0.01 to 0.05; without stars: not significant, P ≥ 0.05. (For interpretation of the references to colour in this figure legend, the reader is referred to the Web version of this article.)

**The medium flow rate is involved in biofilm architecture**. CmpX is a small mechanosensitive channel-like protein, which could be sensitive to mechanical forces. We thus sought to evaluate if the modulation of the flow rate of the liquid medium applied to biofilm cultures grown under dynamic conditions could influence the biofilm architecture and/or cell morphology. In the previous assays, the medium flow rate within the flow cell system was set at 3 mL h^−1^. Here, the biofilm of the H103*-*cmpX and its control H103-EV was allowed to grow in a flow cell with a flow rate of 1.2 or 4.8 mL h^−1^ before CLSM observations and COMSTAT2 analysis. Data corresponding to assays performed at a flow rate of 3 mL h^−1^ were included for comparison purposes. As shown on Fig. 4A, increased biofilm formation seems to be flow rate-dependent. In a first step, we observed the effect of the flow rate on the biofilm formed by the control strain H103-EV. In each case, the biofilm of H103-EV remains flat and homogeneous (Fig. 4A). COMSTAT2 analysis (Fig. 4B, green diagrams) led to show that the biovolume increase was dependent on the applied flow rate, reaching 2.4, 5.7 and 9 μm^3^ μm^−2^ at 1.2, 3, and 4.8 mL h^−1^, respectively. A similar result was observed for the average thickness, reaching 4.9, 7, and 10.2 μm, respectively (Fig. 4C, green diagrams). Maximal thickness of the biofilm was also increased significantly from 9.6 to 15 μm at flow rates of 1.2- and 3-mL h^−1^, respectively. The biofilm thickness reached at 4.8 mL h^−1^ was not significantly different from the one observed at 3 mL h^−1^ (Fig. 4D, green diagrams). Altogether, these data indicate that the flow rate within the flow cell system has a clear involvement on the biofilm architecture characteristics in *P. aeruginosa* H103.

While mostly similar, the effects of the flow rate on the biofilm architecture of the *cmpX* overexpressing strain, display however some differences. The biofilm formed by H103*-*cmpX strain remains heterogeneous whatever the imposed flow rate (Fig. 4A). As for the H103-EV strain, biofilm formed at a flow rate of 3 mL h^−1^ is greater than that formed at 1.2 mL h^−1^, in terms of biovolume and average thickness, but also in maximal thickness (Fig. 4B, C, 4D, orange diagrams). The biofilms formed at a flow rate of 3 mL h^−1^ and 4.8 mL h^−1^ did not lead to any variation in biovolume or average thickness, but in enhanced maximal thickness, suggesting an increased heterogeneity of the biofilm architecture (Fig. 4D, orange diagrams). Overall, these data show that the flow rate of the medium used under dynamic conditions contributes to the alteration of biofilm architecture in *P. aeruginosa* overexpressing *cmpX* strain.

**Fig. 5:**
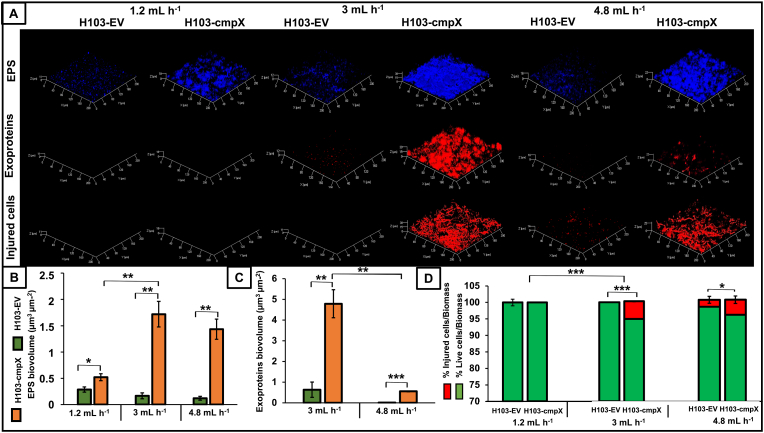
**Effect of flow rates on matrix components.** CLSM images 3D of 24 h biofilms after EPS labelling by calcofluor white (blue), matrix proteins by SYPRO Ruby (red) and injured cells by propidium iodide (red) at 1.2 mL h^−1^, 3 mL h^−1^ and 4.8 mL h^−1^ in H103-EV and H103*-*cmpX. Images show representative data from three independent biofilm assays **(A)**. COMSTAT2 analyses were performed from CLSM images to determine biovolume of EPS **(B)**, and matrix proteins **(C)** of biofilms formed by H103-EV (green) and H103*-*cmpX (orange). No matrix protein production was observed at 1.2 mL h^−1^. The error bars represent the standard error of the means (SEMs) and are the result of the analysis of three views of each of the three independent biological assays obtained by CLSM assays. COMSTAT2 analyses from CLSM images were performed to determine biovolume of live cells (green bars) or injured cells (red bars). The error bars represent the standard error of the means (SEMs) and are the result of the analysis of three views of each of the three independent biological assays **(D)**. Statistics were achieved by student's *t*-test between H103-EV and H103*-*cmpX. ***, P = 0.0001 to 0.001; **, P = 0.001 to 0.01; *, P = 0.01 to 0.05; without stars: not significant, P ≥ 0.05. (For interpretation of the references to colour in this figure legend, the reader is referred to the Web version of this article.)

**The medium flow rate modulates the composition of the biofilm matrix and is involved in cell damage within the biofilm.** We next assayed the matrix composition of H103-EV and H103*-*cmpX biofilms grown under dynamic conditions at 1.2, 3 or 4.8 mL h^−1^ medium flow rates, using differential fluorescent labels, *i.e*., CFW for EPS, DDAO for eDNA, Sypro Ruby for proteins. Representative CLSM images are shown on Fig. 5A, and COMSTAT2 analysis of the CLSM images on Fig. 5B and C. No significant variation in terms of EPS and exoproteins abundance were observed by COMSTAT2 analysis in H103-EV biofilm matrix (Fig. 5A, B, 5C). In H103*-*cmpX biofilms, the EPS abundance was strongly increased by about 3-fold between the flow rates of 1.2 and 3 mL h^−1^ (Fig. 5A and B). These results are consistent with the biofilm increase observed in these conditions (Fig. 4). DDAO labeling (eDNA) was too low to be measured at 1.2 and 4.8 mL h^−1^ in both strains (data not shown), leading to the absence of COMSTAT2 data at these flow rates. Exocellular proteins abundance was higher at a flow rate of 3 mL h^−1^ compared to 4.8 mL h^−1^ in both strains (Fig. 5C), and could not be evaluated at a flow rate of 1.2 mL h^−1^. In addition, both EPS and exoproteins abundances were significantly higher in H103*-*cmpX strain compared to H103-EV control strain at each flow rate evaluated (Fig. 5). Altogether, these data suggest an involvement of the flow rate in matrix composition of biofilms, a phenotype that was exacerbated in H103*-*cmpX strain compared to H103-EV.

We next questioned the effect of the medium flow rate on the permeability of H103-EV and H103*-*cmpX strains within biofilm structures using the LIVE/DEAD assay (Fig. 5D). We observed an alteration in cell permeability in H103-cmpX with a flow rate of 3 mL h^−1^ compared to 1.2 mL h^−1^, with 6 % of permeabilized cells to the red propidium iodide fluorescent dye, and 94 % of alive cells (Fig. 5D). There is also a tendency to increase the proportion of injured H103-EV cells when flow rates of 3 or 4.8 mL h^−1^ were applied that remains not significant, since COMSTAT2 analysis (Fig. 5D) indicated about 2 % of permeabilized cells and 98 % of alive cells. By contrast, the H103*-*cmpX cells are similarly altered at flow rates of 3 and 4.8 mL h^−1^, the difference between both flow rates being not significant (Fig. 5D). Overall, these data suggest that H103*-*cmpX cells are more sensitive to mechanical forces induced by the flow rate as compared to the corresponding control strain.

**Fig. 6:**
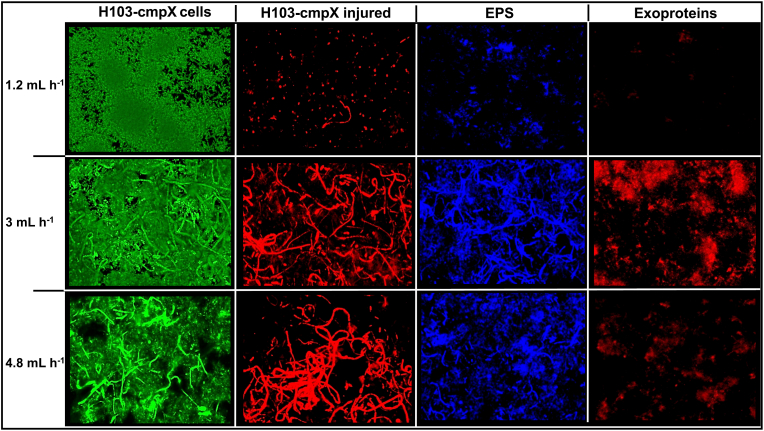
**Effect of flow rates on cell morphology.** Focus of cell morphology observed by CLSM (2D-images) within 24 h biofilms of H103*-*cmpX strain grown in LB with 0.2% arabinose at flow rates of 1.2 mL h^−1^, 3 mL h^−1^ and 4.8 mL h^−1^. Biomass was labeled by SYTO 9 (green), injured cells by propidium iodide (red), EPS by CFW (blue) and exoproteins by Sypro Ruby (red). Images show representative data from three independent biofilm assays. (For interpretation of the references to colour in this figure legend, the reader is referred to the Web version of this article.)

**The flow rate of the medium is involved in *cmpX***^**+**^**cells filamentation.** As indicated earlier, we noticed that cell morphology was strongly altered in the H103-cmpX strain grown under dynamic conditions at a flow rate of 3 mL h^−1^, since numerous single and elongated thin cells were observed (Fig. 2A). We then assayed the effect of the flow rate on the appearance of filamentous cells (Fig. 6). No such cells were detected in H103-EV biofilms, whatever the flow rate imposed (Fig. 4A). CLSM images of H103*-*cmpX biofilms shows that few elongated cells were observed when biofilms were grown at flow rate of 1.2 mL h^−1^ (Fig. 6). On the opposite, these filamentous cells were clearly present when flow rates of 3 and 4.8 mL h^−1^ were applied to biofilms cultures of H103-cmpX. CFW was at least partly associated to the filamentous cells at a flow rate of 3 mL h^−1^ (Fig. 6). Exoproteins seems not to be associated to elongated cells, but were forming some aggregates within biofilm, which are more present at 3 mL h^−1^ compared to 4.8 and 1.2 mL h^−1^. Altogether, these data suggest a relation between *cmpX* overexpression and cell filamentation occurrence in response to the medium flow rate.

## 3 Discussion

**Fig. 7:**
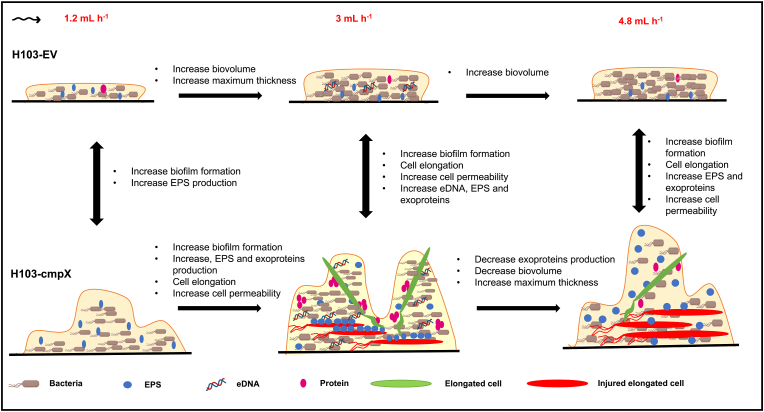
**Schematic view representing the potential effects of *cmpX* overexpression and medium flow rates variations.** Increased flow rate leads to increased biofilm formation in H103-EV. A flow rate of 3 mL h^−1^ causes an increase in biofilm formation and the production of matrix components, as well as cell filamentation compared to a flow rate of 2 mL h^−1^ in *cmpX* overexpression strain. The elongated cells at the base of the biofilm are severely altered, compare to those at the top. Overall, *cmpX* overexpression leads to increase biofilm formation, cell permeability, cell elongation and matrix components production compared to H103-EV under dynamic conditions.

CmpX was previously associated to biofilm alterations, since the deletion of *cmpX* was shown to increase biofilm formation, a phenotype that was restored by *trans*-complementation [25]. This previous study of Bhagirath et al. [25] was conducted in static conditions on polystyrene microtiter plates using the crystal violet staining assay. Herein, we choose to investigate the effects of CmpX in dynamic flow cell systems, on glass slides to be observed by CLSM, since biofilms are involved in chronic infections, noticeably in case of respiratory and urinary infections in which cells are subjected to liquid flow rates. We show that overexpression of *cmpX* in *P. aeruginosa* leads to enhanced and altered biofilm architecture that seems to be associated to increased matrix components and the emergence of filamentous cells under dynamic condition. These phenotypic alterations might occur potentially through a shear stress induced by the medium flow rate. The main results of this study are depicted on Fig. 7.

***cmpX* overexpression affects biofilm architecture and matrix components abundance under dynamic condition with a flow rate of 3 mL h**^**−1**^**.** Herein, we show that *cmpX* overexpression leads to increased biofilm formation in terms of biovolume, average and maximal thicknesses, in dynamic conditions by using a flow rate of 3 mL h^−1^, compared to H103-EV in the same conditions (Fig. 7, middle of the panel). This phenotype was associated to a significant increase in the abundance of EPS, exoproteins and eDNA, according to their role as major actors of the scaffold of the biofilm matrix [[33], [34], [35]]. Since Pel, Psl and alginates are the major EPS of *P. aeruginosa* PAO1 biofilm matrix [36], the transcript levels of *pelB, pslB* and *algD* were quantified by RT-qPCR in biofilm-embedded cells formed with a flow rate of 3 mL h^−1^. While *pslB* and *algU* mRNA levels were unaffected in H103-cmpX compared to H103-EV, expression of *pelB* was upregulated in H103*-*cmpX strain, suggesting that increased Pel production could be responsible for EPS content enhancement. Since the expression of *pel* and *psl* is stimulated by high intracellular c-di-GMP levels [[36], [37], [38], [39]], we next assayed expression of the c-di-GMP-dependent *cdrA* expression [40], and of the DGC PA1181, both expression of which was increased in the H103*-*cmpX strain, suggesting an increase in the c-di-GMP pool. Another study has shown that c-di-GMP produced by the diguanylate cyclase WspR specifically affects Pel but not Psl synthesis [41]. It is thus possible that WspR activity was enhanced in H103*-*cmpX strain in our conditions. CdrA is a fibrillar-like adhesin [32], which links to Psl to contribute to biofilm stability [39], to Pel to promote bacterial aggregation [42], and through CdrA-CdrA interactions [35]. As an exoprotein, CdrA may contribute to the large SYPRO Ruby labelling that was observed in the H103*-*cmpX strain compared to H103-EV. Finally, overexpression of *cmpX* leads to increase the proportion of injured cells within the biofilm, which may explain the significant eDNA abundance, since the release of eDNA is enabled by cell lysis within the biofilm [43]. Our results suggest that CmpX is involved in the production of matrix components which in turn results in increased biofilm formation and altered architecture in dynamic conditions, with a flow rate of 3 mL h^−1^ but also 1.2 and 4.8 mL h^−1^ (Fig. 5, Fig. 6), thus contributing to strengthening the biofilm through a molecular mechanism that remains to be deciphered.

**Effect of the flow rate on the biofilm architecture and matrix components.** We also evaluated the effect of the medium flow rate on *P. aeruginosa* H103-EV and H103*-*cmpX strains at the levels of biofilm biomass, architecture, and of matrix components abundances. Biofilms formed by the H103-EV strain at a flow rate of 1.2, 3, or 4.8 mL h^−1^ show increased biofilm formation, in terms of biovolume, which remains however homogeneously flat. While the average thickness was significantly increased when biofilms are formed at a flow rate of 4.8 mL h^−1^ compared to a flow rate of 3 mL h^−1^, this was not the case between 1.2 and 3 mL h^−1^, despite a tendency to increase. The maximal thickness was significantly increased when biofilms are formed at 3 mL h^−1^ compared to 1.2 mL h^−1^, but nothing seems to happen for higher flow rates. Noticeably, we observed no significant matrix components abundance differences with regards to the flow rate for the H103-EV control strain. Finally, biofilms formed at 4.8 mL h^−1^ led to an increase cell permeability that remains however, not significant compared to a flow rate of 3 mL h^−1^. Altogether, our data suggest that *P. aeruginosa* is sensitive to the medium flow rate that seems to influence biofilm formation. Since biofilm is defined as a protective structure [45], the mechanical shear stress generated by the increased flow rate is perceived by the cells, which in turn could lead to induced biofilm formation. The effect of shear stress on biofilm formation was previously investigated. An exponential and asymptotic decrease of the biofilm thickness and mass was observed with increasing shear stress. On contrary bacterial density increased with shear stress [46]. In addition, the fluid flow was recently shown to mediate the spatial organization of microbiota communities [47]. However, the molecular mechanisms underlying this phenotype are still unclear.

By contrast, H103*-*cmpX showed an exacerbated phenotype compared to H103-EV, with a maximum biofilm increase in terms of biovolume and average thickness that was observed at a flow rate of 3 mL h^−1^. Such modifications were associated to enhanced EPS and exoproteins abundance within the biofilm matrix that was maximal at 3 mL h^−1^, according to increased expression of gene, encoding proteins involved in EPS or exoproteins, as indicated above. As mechanosensitive ion channels are membrane tensions sensors [48], it is possible that increasing *cmpX* expression results into an amplified response to flow rate variations. In addition, H103*-*cmpX showed a significant biofilm heterogeneity in response to flow rate variations, suggesting that CmpX is involved in biofilm architecture in these conditions (Fig. 7). Indeed, the maximal thickness of biofilms formed at 3 mL h^−1^ increased proportionally according to the flow rate from 1.2 to 4.8 mL h^−1^ in H103-cmpX. It is possible that this biofilm enhancement could help to limit the effects of the shear stress generated by the flow rate. Otherwise, it is also possible that the shear stress induced by the flow rate would provoke such heterogeneity. Altogether, our results suggest that CmpX is involved in the production of matrix components in response to mechanical stresses applied by the medium flow, contributing thus to strengthening the biofilm to its constraints.

**Elongated cells abundance increased in H103*-*cmpX with regards to the medium flow rate.** Another major result of this study is the emergence of elongated cells within the biofilm of the H103*-*cmpX overexpressing strain with regards to the medium flow rate (Fig. 7). Cell filamentation is generally associated with cellular stress [49], suggesting that the mechanical forces exerted by the flow may be perceived as a stimulus when *cmpX* is overexpressed. Their presence at the top of the biofilm could contribute to the heterogeneity due to the increase of the maximum thickness observed in the H103*-*cmpX. By contrast to the cells located at the top of the biofilm, the elongated cells situated at the biofilm basis are altered, as shown by CLSM image observations. This phenotype can be associated to increased cell injured with regards to the flow rate, which is consistent with the release of cellular content such as nutrients, or eDNA that could help to strengthen the bottom part of the biofilm in response to flow. The association of these elongated cells with the EPS could also help to strengthen the biofilm by binding the EPS to the other matrix components (proteins and eDNA), thereby increasing matrix cross-linking. Filamentation has been often associated to cell division inhibition [50]. Cell division is performed at least partly by the tubulin homologue FtsZ that is a major player of the divisome [51]. As shown in our study, *ftsZ* expression was significantly increased in H103*-*cmpX strain compared to H103-EV at 3 mL h^−1^ (Table 1). Our results are contradictory with data from the literature where increased *ftsZ* expression is associated to ovoid cells [51,52]. It is possible that the production or the activity of FtsZ, as well as the activity of the proteins involved in its degradation may be altered. On the other hand, cell filamentation could result from a destabilization of H103*-*cmpX envelope upon the mechanical stresses generated by the flow. Indeed, in conditions of envelope alterations such as absence of *oprF* encoding the structural porin OprF [20,44] or infection with the filamentous phage Pf4 [24], biofilm formation was shown to be increased, as well as gene expression of *cmpX,* PA1181, *cdrA,* and *pel,* but not *psl* [20,24]. Infection with Pf4 also resulted in cell filamentation. These conditions have been previously associated to the activation of the CESR ECFσ SigX [24,44] and suggest that overexpressing *cmpX* would induce a CESR. In our conditions, however, we did not observe any expression alteration of *algU* and *sigX* envelope stress response ECFs, as well as their respective *algD* and *cfrX* targets in H103*-*cmpX. However, the increased expression of PA1181, encoding a diguanylate cyclase that may be a member of the SigX operon [20], suggest a partial increase of SigX activity. The localization of *cmpX* upstream of *sigX* and the involvement of SigX in its transcription suggest that CmpX could be part of the CESR [11]. In addition, the *E. coli* MscS mechanosensitive properties were recently shown to be affected by membrane stiffness [53].

Altogether these data suggest that the MSC CmpX would have an unsuspected new function as a membrane sensor of an atypical original signal transduction system that responds to membrane structure alterations in *P. aeruginosa.* Further experiments aiming at investigating the role of CmpX in membrane homeostasis would now be of interest.

## 4 Experimental procedures

**Bacterial strains, growth conditions and monitoring.** The strains used in this study are listed in Supplementary Table S1. *P. aeruginosa* H103-EV and H103*-*cmpX were grown in Luria-Bertani broth containing 171 mM (10 g L^−1^) NaCl (LB) with 0.2% of arabinose. *P. aeruginosa* H103-EV and H103*-*cmpX was grown overnight (18 h) in LB at 37 °C with shaking (180 rpm), and tetracycline 100 μg mL^−1^ was used to grow H103*-*cmpX. Then a cell suspension of each strain at A_580nm_ of 0.08 was grown in LB medium with 0.2% of arabinose for 24 h at 37 °C with shaking (180 rpm) in 96-well microtiter plates. An absorbance measure was performed every 30 min using the Spark 20 M multimode Microplate Reader controlled by Spark Control™ software Version 2.1 (Tecan Group Ltd., Crailsheim, Germany). The data were plotted, and each point indicates the mean ± standard deviation (SD) of A_580nm_ values.

**General DNA procedures.** Restriction enzymes, T4 DNA ligase, and alkaline phosphatase were purchased from New England Biolabs (Ipswich, MA) and used according to the manufacturer's instructions. PCR assays were carried out with 1 μg of *P. aeruginosa* strain H103 chromosomal DNA, 20 pmol of each primer and Taq DNA polymerase (Roche Molecular Biochemicals). When necessary, PCR products and plasmids were purified with the QIAquick or QIAprep Spin Miniprep kits (QIAGEN), respectively. *E. coli* and *P. aeruginosa* were transformed by electroporation (Gene Transformer GTF100, Savant) as previously described [54,55].

**Construction of the *cmpX*-supplemented wildtype strain and its control strain**. To supplement H103 wildtype strain with a copy of *cmpX*, the mini-CTX1-araC-pBAD was used [20]. The *cmpX* gene (PA1775) was amplified by PCR using the primer pair ScmpXmini (TAATAAAAGCTTCATCTTCTGCTGACAAGGTGAGTG) and AScmpXmini (TAATAAGTCGACTTATCTCTCCCGGATCAGCG). The PCR product was digested with *Hind*III and *Sal*I and ligated into the *Hin*dIII-*Sal*I digested mini-CTX1-araC-pBAD vector to create the mini-CTX1-araC-pBAD-cmpX. The sequence of this construct was verified by DNA sequencing using the primer OAL571 (CGGCGTCACACTTTGCTATG). This vector was constructed in *E. coli* JM109 strain, purified and transferred into *E. coli* SM10 strain. The mini-CTX1-araC-pBAD-cmpX vector as well as the native mini-CTX1-araC-pBAD vector were mobilized from *E. coli* SM10 into H103 by conjugation to generate the CmpX overproducing strain H103-cmpX, and its control isogenic strain H103-EV (empty vector). Transconjugates were selected onto PIA agar plate containing 250 μg mL^−1^ of tetracycline. The insertion was verified by PCR using the primer OAL571 and sequencing.

**Flow cell biofilm assays under dynamic conditions.** The flow cell system, which allows continuous bacterial biofilm formation, was assembled, prepared, and sterilized as described by Tolker-Nielson and Sternberg [56]. Bacterial cells from an overnight culture, were washed 3 times before being adjusted at an A_580_ of 0.1 in sterile physiological water (0.9% w/v NaCl) to remove putative residual tetracycline. Each channel of the flow cell (1 mm × 4 mm x 40 mm Bio centrum, DTU, Denmark) was inoculated with 300 μL of the bacterial suspension. Bacterial adhesion was performed without any flow for 2 h at 37 °C. After 2 h of adhesion, LB containing 0.2% arabinose for H103*-*cmpX and H103-EV was pumped with a flow rate of 1.2, 3 or 4.8 mL h^−1^ at 37 °C for 24 h.

**Adhesion assays.** Cells from an overnight culture were washed and adjusted at an A_580nm_ value of 0.1 in sterile physiological water (0.9% w/v NaCl), before being allowed to adhere for 2 h on a glass slide at 37 °C. After 3 washes with PBS 1X, cells were labeled with 5 μM of SYTO® 9 green fluorescent nucleic acid stain (Invitrogen, Carlsbad, CA), and observed under CLSM.

**Confocal Laser Scanning Microscopy (CLSM).** Prior to image acquisition, biofilm or planktonic cells were labeled with fluorescent dyes and observed by CLSM. Cells were stained by adding 5 μM of SYTO® 9 green fluorescent nucleic acid stain (Invitrogen, Carlsbad, CA). The Live/Dead fluorescent staining was performed using the LIVE/DEAD™ BacLight™ Bacterial Viability Kit (Thermofisher®). Briefly, cells were labeled with a mixture (v/v) of component A (SYTO 9, 1.67 mM/propidium iodide, 1.67 mM) and component B (SYTO 9, 1.67 mM/propidium iodide 18.3 mM) according to the recommendations of the supplier. Extracellular DNA was detected using 1 μM of 7‐hydroxy‐9H‐(1,3‐dichloro‐9,9‐dimethylacridin‐2‐one) (DDAO, Invitrogen, Carlsbad, CA), and exopolysaccharides by 200 μg mL^−1^ of CalcoFluor White M2R (CFW, Sigma‐Aldrich, USA). Matrix proteins were labeled by 350 μL of Sypro Ruby (Invitrogen, Carlsbad, CA), injected directly into the flow cell channel. The CLSM observations were carried out with a Zeiss LSM710 (Carl Zeiss Microscopy, Oberkochen, Germany) using a 40× oil immersion objective. Images were taken every micrometer throughout the whole biofilm depth. For visualization and processing of three-dimensional (3D) images, the Zen 2.1 SP1 zen software (https://www.zeiss.com/microscopy/int/downloads/zen.html) (Carl Zeiss Microscopy, Oberkochen, Germany) was used. The average and maximum thicknesses (μm) and biovolumes (μm^3^ μm^−2^) of biofilms were measured using the COMSTAT software (http://www.imageanalysis.dk/) [57]. At least three image stacks from at least three independent experiments were used for each analysis.

**RNA extraction and quantitative RT-PCR.** Total RNAs from three independent biofilm cultures were isolated by the hot acid-phenol method as previously described [21], with minor modifications. Biofilms were grown in the flow cell system as described above. After 24 h, 500 μL of the lysis buffer was injected in each channel and after back-and-forth movements, the suspension was recovered at the channel outlet. Rigorous treatments with Turbo DNA-*free*™ kit (Invitrogen) were then performed according to the manufacturer. Synthesis of cDNAs and RT-qPCR were achieved as previously described [58], using the primers listed in Supplementary Table S2. The expression level of the mRNAs or sRNAs was calculated by comparing threshold cycle (Ct) of target genes with control sample group and the relative quantification data was determined with the 2^−ΔΔCt^ method [59] using DataAssist™ software (Applied Biosystems).

**Statistical analysis.** Statistical significance was evaluated using Prism GraphPad online tool (https://www.graphpad.com/quickcalcs/ttest1/). The data were statistically analyzed using two-sample unpaired *t*-test to calculate *p* values. The mean with SD or SEM were calculated and plotted.

## CRediT authorship contribution statement

**Audrey David:** Writing – review & editing, Methodology, Investigation, Formal analysis, Data curation. **Mélissande Louis:** Methodology, Formal analysis. **Ali Tahrioui:** Writing – review & editing, Methodology, Investigation, Formal analysis, Data curation, Conceptualization. **Sophie Rodrigues:** Writing – review & editing, Supervision, Methodology, Formal analysis, Data curation. **Clarisse Labbé:** Methodology, Formal analysis. **Olivier Maillot:** Methodology, Formal analysis. **Magalie Barreau:** Methodology, Formal analysis. **Olivier Lesouhaitier:** Writing – review & editing, Methodology, Formal analysis. **Pierre Cornelis:** Writing – review & editing, Writing – original draft, Data curation, Conceptualization. **Sylvie Chevalier:** Writing – review & editing, Writing – original draft, Validation, Supervision, Resources, Project administration, Investigation, Funding acquisition, Formal analysis, Data curation, Conceptualization. **Emeline Bouffartigues:** Writing – review & editing, Validation, Supervision, Methodology, Investigation, Formal analysis, Data curation, Conceptualization.

## Declaration of competing interest

The authors declare that they have no known competing financial interests or personal relationships that could have appeared to influence the work reported in this paper.

## Figures and Tables

**figs1:**
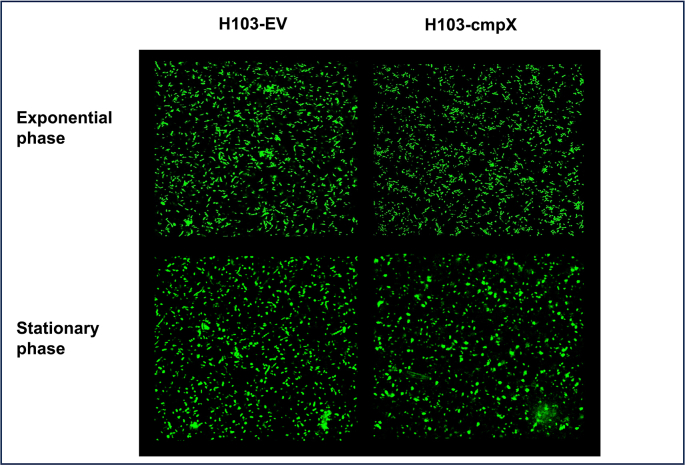


**figs2:**
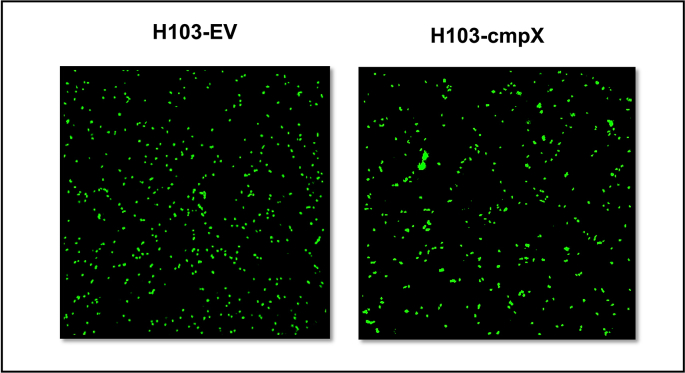


**figs3:**
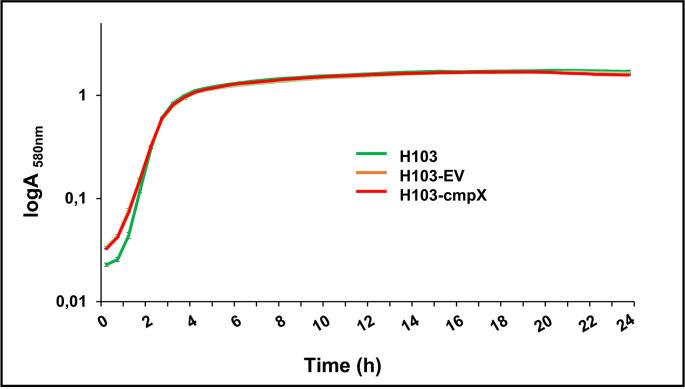


## Data Availability

No data was used for the research described in the article.
