# Supplementary material for: cmpX overexpression in Pseudomonas aeruginosa affects biofilm formation and cell morphology in response to shear stress
Source: Biofilm. 2024 Mar 15;7:100191. doi: 10.1016/j.bioflm.2024.100191 (PMC10965496; doi:10.1016/j.bioflm.2024.100191)
Supplement: Multimedia component 1 [file mmc1.docx]

**Supplementary Tables**

**Supplementary Table S1**. List of strains used in this study

**Strain Characteristics Reference**

H103 Wild-type strain H103; PAO1 prototroph derivative Hancock et Carey, 1979

H103-cmpX H103 strain with chromosomal overexpression This study

of *cmpX* and a tetracycline resistance gene;

arabinose inducible

**Supplementary Table S2**. List of primers used in this study

| PA number | Gene name | Primer name | Sequence (5’ - 3’) |
| --- | --- | --- | --- |
| PA0762 | *algU* | F-algU  R-algU | TACCTGGCTGTATCGGATCG |
|  |  |  | GAAGAACTCCGCATCCTCTG |
| PA3540 | *algD* | F-algD  R-algD | GGGCTATGTCGGTGCAGTATG |
|  |  |  | GCGACTTGCCCTGGTTGAT |
| PA1776 | *sigX* | F-sigX  R-sigX | AATTGATGCGGCGTTACCA |
|  |  |  | CCAGGTAGCGGGCACAGA |
| PA1774 | *cfrX* | F-sigX  R-sigX | CTGCGGGACCTCGTCAAG |
|  |  |  | GCCGACCTGGCGATTG |
| PA1775 | *cmpX* | F-cmpX  R-cmpX | TATCTGGACCCAGAGCTTGC |
|  |  |  | GAAGCCGAGCAGAACGAC |
| PA2232 | *pslB* | F-pslB  R-pslB | ACACCAACGAATCCACCTTCA |
|  |  |  | CGCTCTGTACCTCGATCATCAC |
| PA3063 | *pelB* | F-pelB  R-pelB | CGGCTACGTGCAGCGTTAT |
|  |  |  | CACTGCATGCGTTCCTTGAC |
| PA3621.1 | *rsmZ* | F-rsmZ  R-rsmZ | CGTACAGGGAACACGCAAC |
|  |  |  | TTCAGTCCCTCGTCATCATC |
| PA4570.1 | *rsmW* | F-rsmW  R-rsmW | CTGAGCCTTTGCGATGACGC |
|  |  |  | GCCTTTGCCTCCGTTCGTGCGATC |
| PA0527.1 | *rsmY* | F-rsmY  R-rsmy | CGCCAAAGACAATACGGAAAC |
|  |  |  | TTTTGCAGACCTCTATCCTGACATC |
| PA4407 | *ftsZ* | F-ftsZ  R-ftsZ | AAACCGCCGTCATAAAAGTG |
|  |  |  | GATGTTCTTCAGCGCTTGTG |
| PA4625 | *cdrA* | F-cdrA  R-cdrA | GACGCCTACGTCAACAGTCA |
|  |  |  | GTTACCGGTGATCGCGTACT |
| PA1611 | *PA1611* | F-PA1611  R-PA1611 | CATCAACAATCTCGCCAGCG |
|  |  |  | TCATCATCGCCAGGAAGTCG |
| PA1181 | *PA1181* | F-PA1181  F-PA1181 | CCAGATGGAGAAGCGCTACCT |
|  |  |  | CGCTTGCGACTGTCGATATC |
| PA4843 | *PA4843* | F-PA4843  F-PA4843 | CCTGGGCACCGAATTGG |
|  |  |  | CGGCGGACAGGTAGATGATC |
